# LncRNA SNHG9 is downregulated in osteoarthritis and inhibits chondrocyte apoptosis by downregulating miR-34a through methylation

**DOI:** 10.1186/s12891-020-03497-7

**Published:** 2020-08-01

**Authors:** Hongfei Zhang, Jinglian Li, Weiguang Shao, Naipeng Shen

**Affiliations:** 1 Department of arthritis, Affiliated Hospital of Weifang Medical University, No.2428, Yuhe Road, Kuiwen District, Weifang City, Shandong Province 261031 P.R. China; 2 Weifang Medical University, NO.4948 Shengli East Street, Weifang City, Shandong Province 261042 P.R. China

**Keywords:** Osteoarthritis, SNHG9, miR-34a, Chondrocytes, Methylation

## Abstract

**Background:**

Our preliminary RNA-Seq data revealed altered expression of small nucleolar RNA host gene 9 (SNHG9) in osteoarthritis (OA) and its reverse correlation with miR-34a, which can regulate chondrocyte apoptosis in rat OA model. This study was therefore carried out to investigate the potential interaction between SNHG9 and miR-34a in OA.

**Methods:**

A total of 60 healthy volunteers (Control group) as well as 60 OA patients (OA group) were enrolled in this study. Transfections, RT-qPCR, methylation-specific PCR (MSP) and cell apoptosis assay were performed.

**Results:**

We found that SNHG9 was downregulated in OA and its expression was reversely correlated with the expression of miR-34a only across OA samples but not healthy control samples. In chondrocytes from OA patients, overexpression of SNHG9 led to downregulation of miR-34a and increased methylation of miR-34a gene. In contrast, in chondrocytes from healthy controls, overexpression of SNHG9 did not affect the expression of miR-34a and the methylation of miR-34a gene. Cell apoptosis analysis showed that overexpression of SNHG9 led to decreased apoptotic rate of chondrocytes from OA patients but not chondrocytes from the healthy controls through miR-34a.

**Conclusion:**

In conclusion, SNHG9 is downregulated in OA and inhibits chondrocyte apoptosis by downregulating miR-34a through methylation.

## Background

Osteoarthritis (OA) as the most commonly diagnosed joint disease is caused by the breakdown of cartilage in a joint as well as underlying bone [1]. Osteoarthritis mainly affects elderly and causes chronic pain, resulting in disability [2]. Due to the direct medical cause and indirect reduction in productivity, OA now is considered as a major economic burden on the modern society [3]. Pathological changes of OA cannot be reversed. So there is no cure for this disease [4]. Treatment of OA mainly focuses on pain and symptom relief [4]. The development of novel anti-OA therapies is mainly limited by the unclear molecular mechanisms of this disease [5, 6]. Therefore, investigations of the molecular players involved in this disease are of great importance.

Previous studies on the molecular pathogenesis of OA have characterized a considerable number of signaling pathways involved in this disease [7, 8]. Certain pathways, such as the WNT signaling pathway, have been considered as potential targets for the development of targeted therapies for OA [7]. Non-coding RNAs (ncRNAs), such as microRNAs (miRNAs) and long (> 200 nt) ncRNAs (lncRNAs) are not involved in protein synthesis but participate in the regulations of human diseases including OA by regulating gene expression [9]. Certain lncRNAs, such as PVT1, have been proven to participate in OA through multiple ways, such as regulation of cell apoptosis [10]. However, the roles of most lncRNAs in this disease are still unknown. Small nucleolar RNA host gene 9 (SNHG9), a recently identified lncRNA, has been reported to interact with Wnt2 to participate in glioblastoma [11]. It has been established that the WNT signaling is a critical player in OA [7], indicating the potential involvement of SNHG9 in OA. Our preliminary RNA-Seq data revealed the altered expression of SNHG9 in OA and its reverse correlation with miR-34a, which can regulate chondrocyte apoptosis in rat OA model [12]. This study was therefore carried out to investigate the potential interactions between SNHG9 and miR-34a in OA.

## Methods

### OA patients and healthy controls

A total of 60 healthy volunteers (Control group, 40 females and 20 males, age range from 50 to 70 years old, mean age 60.1 ± 4.8 years old) as well as 60 OA patients (OA group, 40 females and 20 males, age range 50 to 70 years old, mean age 60.1 ± 4.8 years old) were enrolled in this study. All the participants were admitted to Affiliated Hospital of Weifang Medical University between August 2017 and August 2019. This study was approved before the admission of participants by the Ethics Committee of aforementioned hospital. All OA patients were diagnosed by X-ray imaging. In addition, blood tests were also performed to exclude other clinical disorders that cause joint pain, such as rheumatoid arthritis. The healthy volunteers were admitted at the physiological center of aforementioned hospital after they received routine physiological exams. The 60 OA patients included 30 cases of knee-affected and 30 cases of hip-affected cases. In addition, 32 cases were at stage 3 (moderate) and 28 cases at stage IV (severe). All patients were newly diagnosed and other clinical disorders were excluded. No treatments were performed before this study. All participants signed the written form informed consent.

### Synovial fluid preparation

Synovial fluid (2.5 ml) extraction was performed on all OA patients from their affected sites (30 cases of knee and 30 cases of hip). To match the OA group, extraction of synovial fluid (2.5 ml) was also performed on healthy controls with 30 cases of knee and 30 cases of hip. All synovial fluid samples were stored in liquid nitrogen before use.

### Chondrocytes and transfections

Chondrocytes collected from healthy adults (402-05A, Sigma-Aldrich) and from OA patients (402OA-05A, Sigma-Aldrich) were included in this study to match the OA patients and healthy controls included in this study. Cell culture medium was composed of 10% FBS and 90% chondrocyte growth medium (PromoCell). Cell culture conditions were 37 °C, 95% humidity and 5% CO_2._ Cells were collected at about 80% confluence to perform transfections. PcDNA 3.1 was used to construct the expression vector of SNHG9 (NCBI Accession: NR_003142.2). Negative control (NC) miRNA (5′-CUGUACGUGCUAGUGCGUGGA-3′) and miR-34a mimic (5′-UGGCAGUGUCUUAGCUGGUUGU-3′) were obtained from Sigma-Aldrich (USA). MiR-34a inhibitor (Cat # HMI0508-5NMOL) and miRNA inhibitor NC (Cat # NCSTUD001) were also purchased from Sigma-Aldrich. Lipofectamine 2000 (Invitrogen) was used to transfect 10^6^ chondrocytes (two types) with 40 nM miR-34a mimic, 40 nM miRNA inhibitor, or 10 nM SNHG9 expression vector following the manufacturer’s instructions. The transfection mixture was incubated for 6 h. Transfection with empty vector, NC inhibitor or NC miRNA was used as the NC group. Untransfected cells were used as the control (C) group. Following experiments were performed at 48 h post-transfection.

### RNA extractions

RNA extractions from chondrocytes and synovial fluid were performed using Trizol (Invitrogen). To retain miRNAs in RNA samples, 85% ethanol was used for RNA precipitation. All RNA samples were digested with gDNA eraser (Takara) at 37 °C for 2 h to remove genomic DNAs before use. RNA concentrations were measured using Thermo Scientific NanoDrop 2000 spectrophotometer. RNA integrity was checked on 5% urine-PAGE gel.

### RT-qPCR

Total RNA reverse transcriptions were performed using the MMLV Reverse Transcriptase (Lucigen) to prepare cDNA samples through the following conditions: 25 °C for 10 min, 55 °C for 30 min and 85 °C for 10 min. With cDNAs as template, qPCR reactions were performed using SYBR Premix Ex TaqTM II (Takara). The expression levels of SNHG9 were measured with GAPDH as the endogenous control. The expression levels of mature miR-34a were measured using the All-in-One™ miRNA qRT-PCR Reagent Kit (Genecopoeia) following the instructions from Genecopoeia. U6 was used as the internal control for miR-34a. Primer sequences were: 5′-GAATCTACGTCACCCGAAAAG-3′ (forward) and 5′-CAAACACGTGGGACAGCCAAG-3′ (reverse) for SNHG9; 5′-GTCTCCTCTGACTTCAACAGC-3′ (forward) and 5′-ACCACCCTGTTGCTGTAGCCA-3′ (reverse) for GAPDH. The forward primer for miR-34a was 5′-TGGCAGTGTCTTAGCT-3′. Universal reverse primer and U6 forward primer were from the kit. PCR conditions were as following: 95 °C for 1 min, followed by 40 cycles of 95 °C for 10 s and 55 °C for 50 s. Three replicate reactions were included in each experiment. Fold changes of gene expression levels were calculated using 2^−ΔΔCt^ method.

### Methylation-specific PCR (MSP)

ISOLATE II Genomic DNA Kit (Bioline) was used to extract genomic DNAs from chondrocytes. DNAs were converted using EZ DNA Methylation-Gold™ kit (ZYMO). Following PCR reactions were performed using Taq 2X Master Mix (M0270, NEB). PCR conditions were as following: 95 °C for 5 min, followed by 40 cycles of 95 °C for 30 s, 55 °C for 30 s and 72 °C for 50 s, and then 72 °C for 10 min. PCR primers were: 5′-GGCCAGCTGTGAGTGTTTC-3′ (forward) and 5′-GGGCCCCACAACGTGCAG-3′ (reverse). MSP primers were: 5′-GGTTAGTTGTGAGTGTTTT-3′ (forward) and 5′-AAACCCCACAACGTACAA-3′ (reverse).

### Cell apoptosis assay

Chondrocytes were harvested at 48 h post-transfection. After that, 50,000 cells were transferred to each well of a 6-well plate, followed by incubation in cell culture medium containing 300 ng/ml lipopolysaccharide (LPS) for another 48 h. Three replicate wells were set for each experiment. Cells were then collected and stained with propidium iodide (PI) and Annexin-V FITC for 20 min in dark. Finally, flow cytometry was used to separate apoptotic cells.

### Caspase-3 activity assay

ELISA assay was used to measure the activity of caspase-3 in cells of each transfection group included in cell apoptosis assay. After treatment with LPS for 48 h, Caspase-3 ELISA assay kit (R&D, Minneapolis, USA) was used to measure caspase-3 activity. OD values were measured by a microplate reader and normalized to C group.

### Statistical analysis

Three biological replicates were included in each experiment and mean values were calculated and used in data analysis. Correlations were analyzed by Pearson’s correlation coefficient. Comparisons between 2 groups were performed by unpaired t test. Comparisons among multiple groups were performed by ANOVA (one-way) combined with Tukey test. *P* < 0.05 was considered as statistically significant.

## Results

### The expression of SNHG9 and miR-34a was altered in OA

**Fig. 1:**
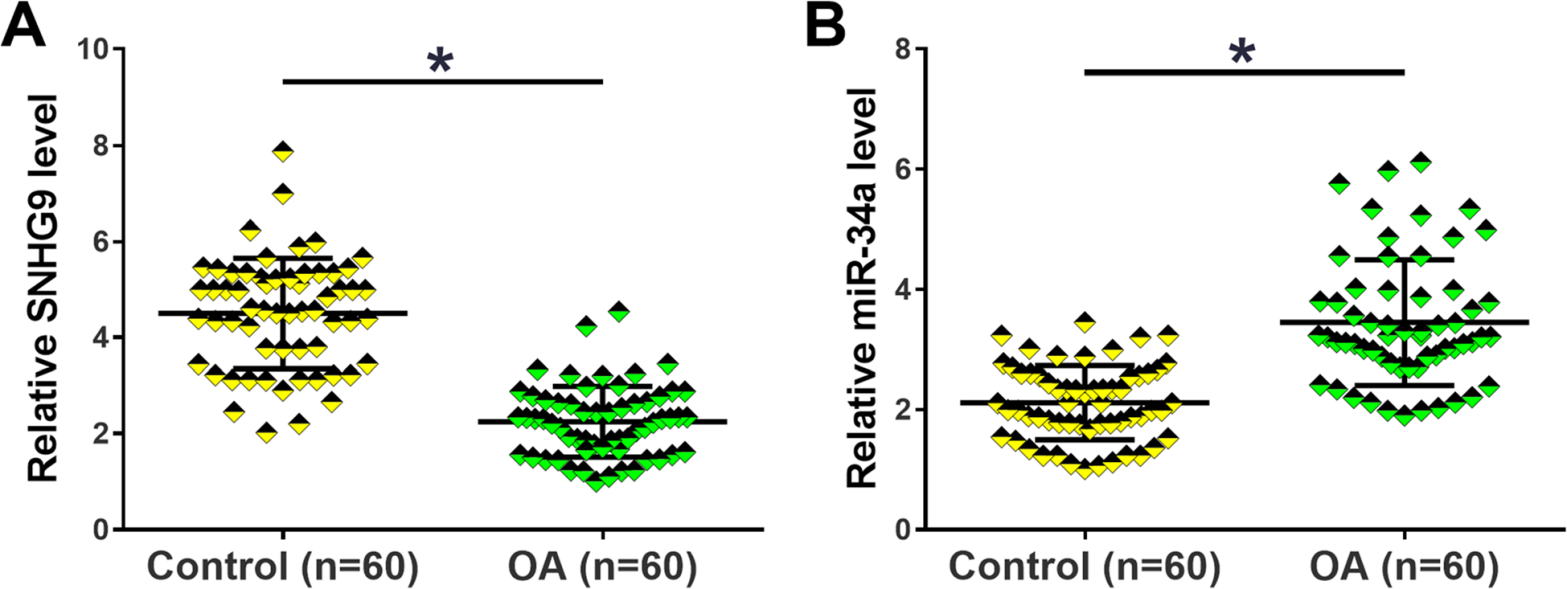
The expression of SNHG9 and miR-34a was altered in OA. Expression levels of SNHG9 (**a**) and miR-34a (**b**) in synovial fluid from both OA patients (*n* = 60) and controls (*n* = 60) were measured using RT-qPCR. PCR reactions were repeated 3 times and mean values were presented and compared. *, *p* < 0.05

Expression levels of SNHG9 and miR-34a in synovial fluid from both OA patients (*n* = 60) and controls (*n* = 60) were measured using RT-qPCR to detect their differential expression in OA. Comparing to the Control group, the expression of SNHG9 was significantly downregulated in OA group (Fig. 1a, *p* < 0.05). By contrast, significantly upregulated miR-34a was observed in OA group in comparison to the control group (Fig. 1b, *p* < 0.05). Therefore, altered expression of SNHG9 and miR-34a may participate in OA.

### SNHG9 and miR-34a were inversely correlated across OA synovial fluid samples

**Fig. 2:**
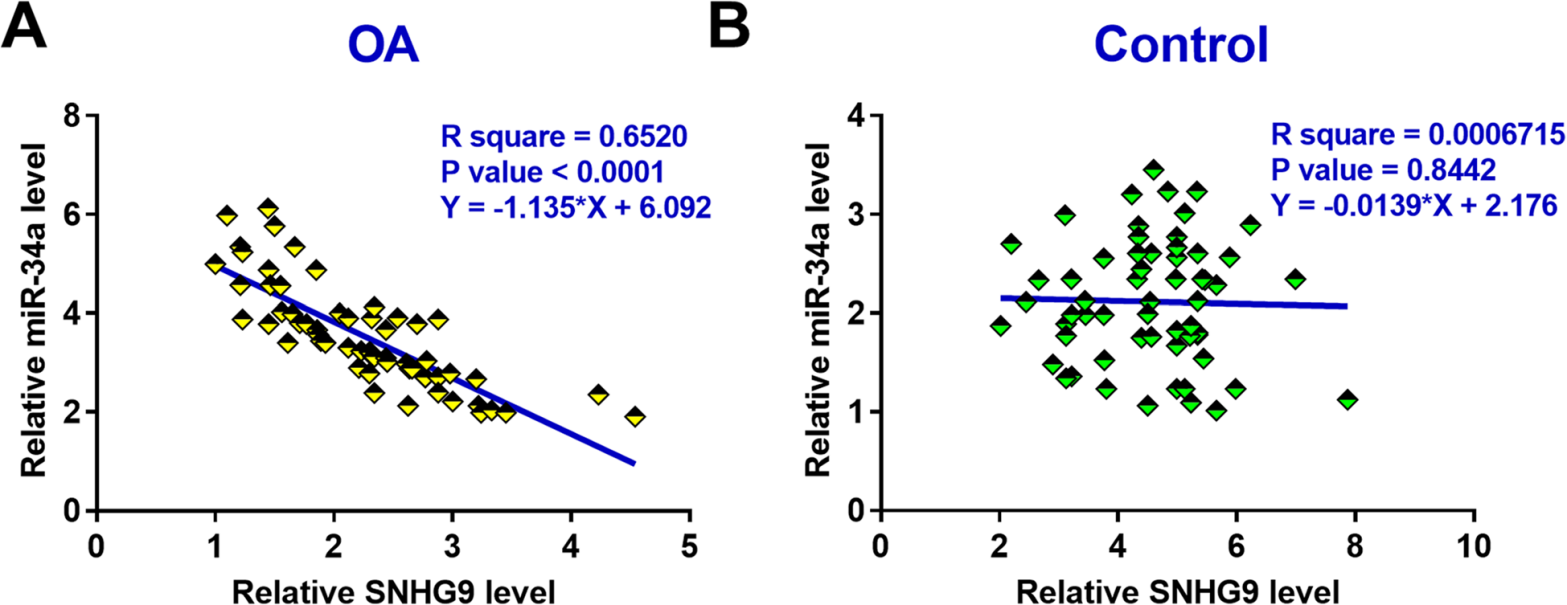
SNHG9 and miR-34a were inversely correlated across OA synovial fluid samples. Correlations between expression levels of SNHG9 and miR-34a across synovial fluid samples from OA patients (**a**) and healthy controls (**b**) were analyzed by Pearson’s correlation coefficient

Correlation analysis revealed that the expression levels of SNHG9 and miR-34a were significantly and inversely correlated across synovial fluid samples from OA patients (Fig. 2a). However, the correlation between the expression levels of SNHG9 and miR-34a was not significantly across synovial fluid samples from healthy controls (Fig. 2b). These data suggested the potential interaction between SNHG9 and miR-34a in OA.

### Overexpression of SNHG9 resulted in the downregulation of miR-34a through methylation in chondrocytes from OA patients

**Fig. 3:**
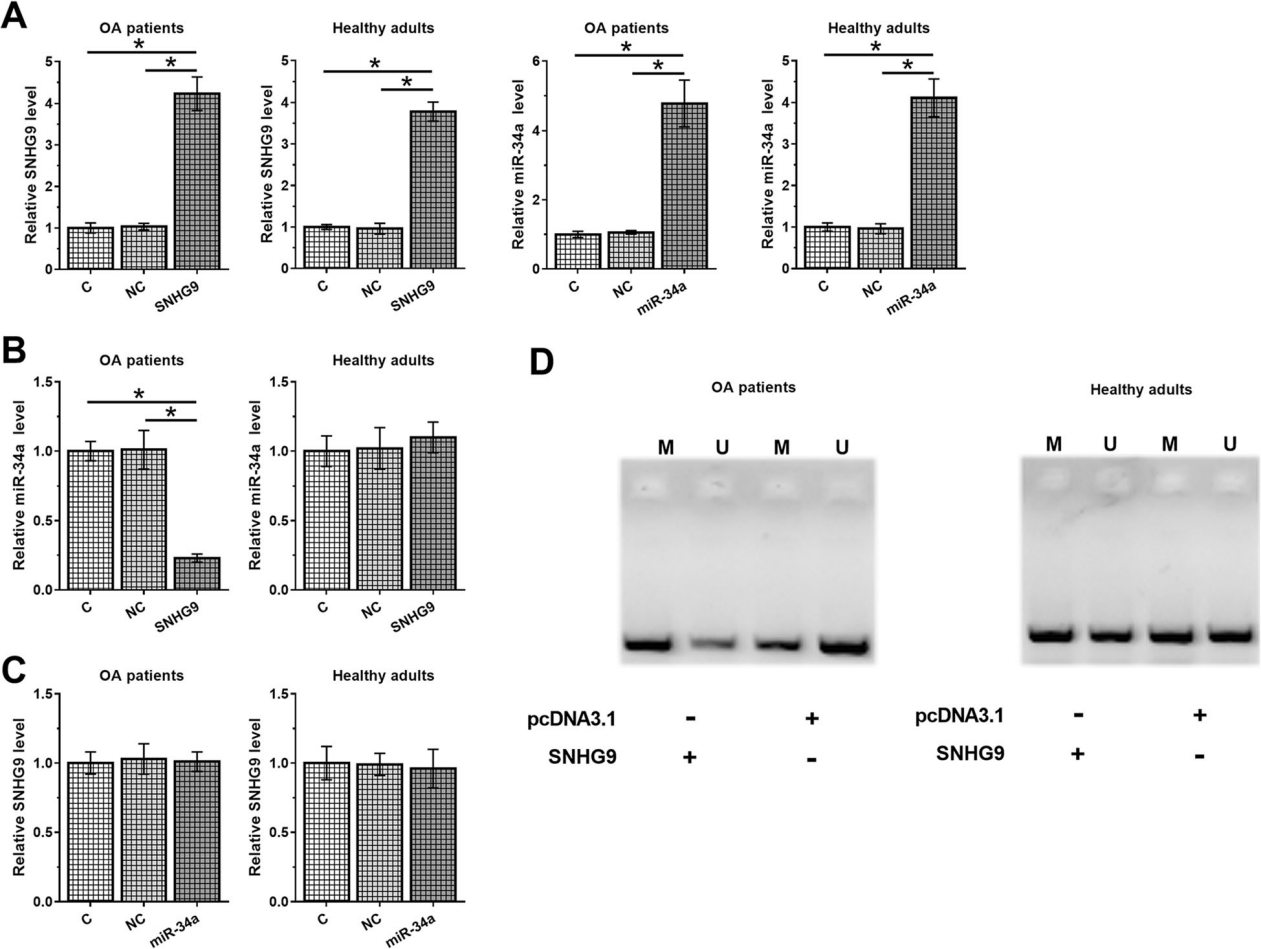
Overexpression of SNHG9 led to the downregulation of miR-34a through methylation in chondrocytes from OA patients. In order to analyze the potential interaction between SNHG9 and miR-34a, chondrocytes from OA patients and healthy controls were transfected with SNHG9 expression vector or miR-34a mimic. Overexpression of SNHG9 and miR-34a in two types of chondrocyte was confirmed at 48 h post-transfection by qPCR (**a**). The effects of overexpression of SNHG9 on miR-34a (**b**) and the effects of overexpression of miR-34a on SNHG9 (**c**) in both types of chondrocyte were analyzed by qPCR. MSP was performed to analyze the effects of overexpression of SNHG9 on the methylation of miR-34a gene in both types of chondrocyte (**d**). All experiments were performed in triplicate manner and mean values were calculated and presented. *, *p* < 0.05

In order to assess the potential interaction between SNHG9 and miR-34a, chondrocytes from OA patients and healthy controls were transfected with SNHG9 expression vector or miR-34a mimic. Overexpression of SNHG9 and miR-34a in two types of chondrocytes was confirmed at 48 h post-transfection by qPCR (Fig. 3a, *p* < 0.05). Comparing to C and NC groups, overexpression of SNHG9 led to the downregulation of miR-34a in chondrocytes from OA patients (*p* < 0.05) but not in chondrocytes from healthy adults (Fig. 3b). Moreover, overexpression of miR-34a did not affect the expression of SNHG9 in both types of chondrocyte (Fig. 3c). MSP was performed to evaluate the effects of overexpression of SNHG9 on the methylation of miR-34a gene in both types of chondrocyte. Comparing to cells transfected with empty vector, overexpression of SNHG9 led to increased methylation of miR-34a gene in chondrocytes from OA patients and but not in chondrocytes from healthy controls (Fig. 3d). As miR-34a is already overexpressed in OA, inhibition of miR-34a in chondrocytes from OA patients was also performed (Supplemental Fig. 1A, *p* < 0.05). It was observed that inhibition of miR-34a did not affect the expression of SNHG9 (Supplemental Fig. 1B).

### Overexpression of SNHG9 led to decreased apoptotic rate of chondrocytes from OA patients through miR-34a

**Fig. 4:**
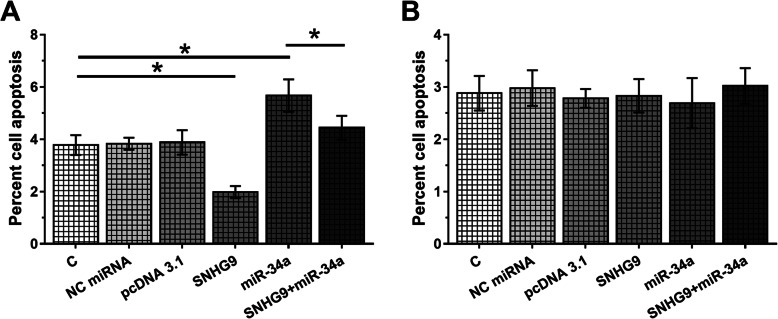
Overexpression of SNHG9 led to decreased apoptotic rate of chondrocytes from OA patients through miR-34a. LPS was used to treat two types of chondrocyte and the effects of overexpression of SNHG9 and miR-34a on the apoptosis of chondrocytes from OA patients (**a**) and healthy controls (**b**) were analyzed by cell apoptosis assay. All experiments were performed in triplicate manner and mean values were calculated and presented. *, *p* < 0.05

**Fig. 5:**
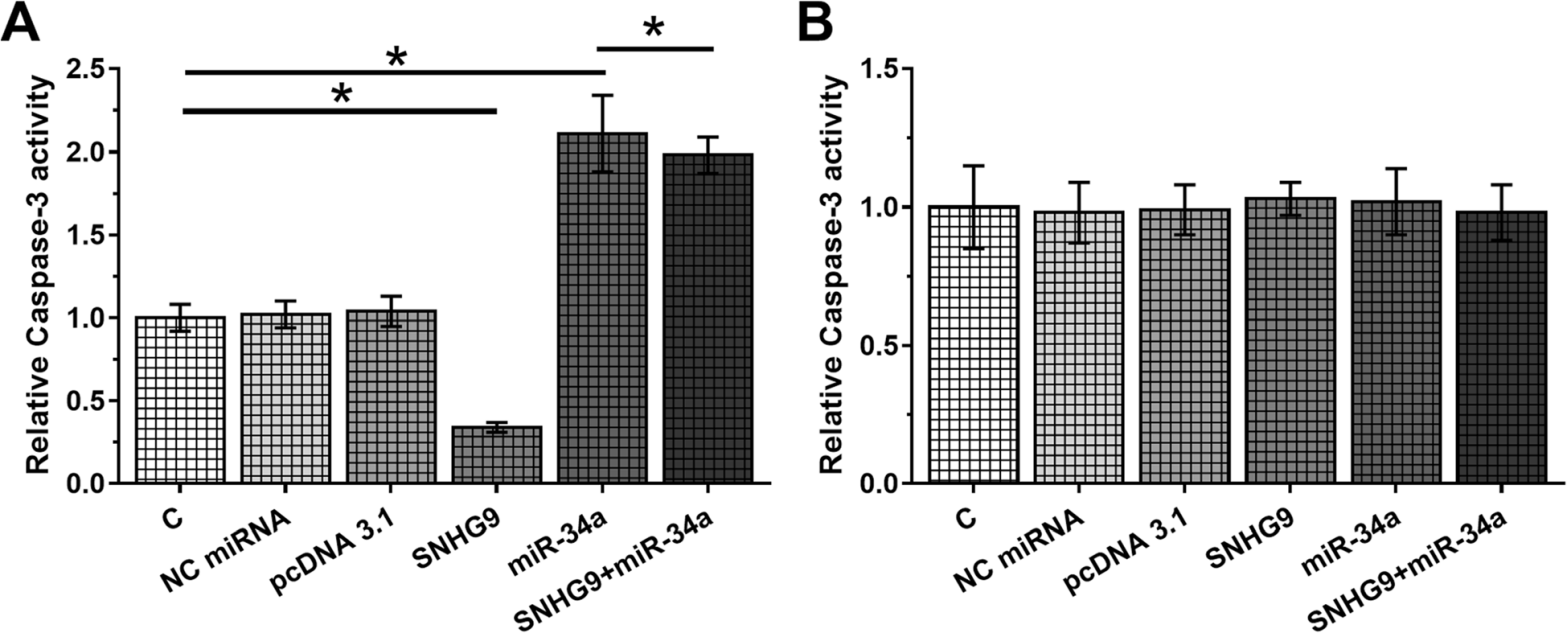
Caspase-3 activity in cell of each transfection groups included in cell apoptosis assay. Caspase-3 activity assay in cell of each transfection groups included in cell apoptosis assay of chondrocytes from OA patients (**a**) and healthy controls (**b**) was measured by ELISA. All experiments were performed in triplicate manner and mean values were calculated and presented. *, *p* < 0.05

LPS was used to treat two types of chondrocyte and the effects of overexpression of SNHG9 and miR-34a on the apoptosis of two types of chondrocyte were evaluated by cell apoptosis assay. Comparing to C and NC groups, overexpression of miR-34a led to increased apoptotic rate of chondrocytes from OA patients (Fig. 4a, *p* < 0.05). Overexpression of SNHG9 played an opposite role and led to inhibited effects of miR-34a (Fig. 4a, *p* < 0.05). In contrast, overexpression of SNHG9 and miR-34a did not affect the apoptosis of chondrocytes from healthy controls (Fig. 4b). Caspase-3 activity in cell of each transfection groups included in cell apoptosis assay was measured by ELISA. It showed that Caspase-3 activity in chondrocytes from OA patients had similar pattern of the cell apoptotic rate (Fig. 5a, *p* < 0.05). In addition, no significant changes in chondrocytes from healthy controls were observed after transfections (Fig. 5b, *p* < 0.05).

## Discussion

To the best of our knowledge, our study is the first to explore the involvement of SNHG9 in OA. We found that SNHG9 was downregulated in OA and may regulate the apoptosis of chondrocytes from OA patients at least partially by downregulating miR-34a through methylation.

The functionality of glioblastoma has been investigated only in glioblastoma [11]. SNHG9 is overexpressed in glioblastoma and upregulate Wnt2 by downregulating miR-199a-5p to promote the proliferation of cancer cells [11]. In another study, the upregulation of SNHG9 was reported to be a potential biomarker for pancreatic cancer [13]. It has been well established that the activation of the WNT signaling in OA promote disease development by promoting articular chondrocyte reprogramming, which leads to the loss of tissue structure [14]. Interestingly, our study showed that SNHG9 was downregulated in OA. As SNHG9 can promote the expression of Wnt2 in glioblastoma [11], we speculated that downregulation of SNHG9 may also lead to inactivation of the WNT signaling in OA. Future studies are needed to evaluate the interaction between SNHG9 and the WNT signaling in OA.

Chondrocytes are the only cells that build up healthy cartilage and the abnormally accelerated chondrocyte apoptosis contributes to the progression of OA [15]. Therefore, inhibition of chondrocyte apoptosis is considered as a potential target for the treatment of OA [16]. In a recent study, miR-34a was reported to promote chondrocyte apoptosis in rat OA model [12]. Consistently, our study showed that miR-34a could promote the apoptosis of chondrocytes from OA patients. Interestingly, our study observed the downregulation of miR-34a by SNHG9 through methylation specifically in chondrocytes from OA patients but not in chondrocytes from health adults. Therefore, SNHG9 may interact with certain pathological mediators to regulate the methylation of miR-34a gene to achieve specific regulation of OA-affected chondrocyte apoptosis. In addition, overexpression of miR-34a also did not affect the apoptosis of the control chondrocytes. Therefore, the role of miR-34a in regulating the apoptosis of chondrocytes in OA patients may also be mediated by certain pathological factors. LPS was used to treat all cells in cell apoptosis assay. Therefore, LPS was excluded from these potential mediators. However, other mechanisms may exist and more studies are needed.

## Conclusion

In conclusion, SNHG9 is downregulated in OA and may downregulate miR-34a expression through methylation to inhibit chondrocyte apoptosis in OA.

## Supplementary information

**Additional file 1: Figure S1.** Inhibition of miR-34a did not affect the expression of SNHG9 in chondrocytes from OA patients. Inhibition of miR-34a in chondrocytes from OA patients was also performed (A). Inhibition of miR-34a also did not affect the expression of SNHG9 (B). All experiments were performed in triplicate manner and mean values were calculated and presented. *, *p* < 0.05.

**Additional file 2: Figure S2.** Full gel of Fig. 3d

## Data Availability

The datasets used and/or analyzed during the current study are available from the corresponding author on reasonable request.

## Abbreviations

SNHG9: Small nucleolar RNA host gene 9
OA: Osteoarthritis
ncRNAs: Non-coding RNAs
miRNAs: microRNAs
lncRNAs: long (> 200 nt) ncRNAs
LPS: Lipopolysaccharide
MSP: Methylation-specific PCR
NC: Negative control
C: Control
