# Supplementary figures and images for: LncRNA SNHG9 is downregulated in osteoarthritis and inhibits chondrocyte apoptosis by downregulating miR-34a through methylation
Source: BMC Musculoskelet Disord. 2020 Aug 1;21:511. doi: 10.1186/s12891-020-03497-7 (PMC7395373; doi:10.1186/s12891-020-03497-7)

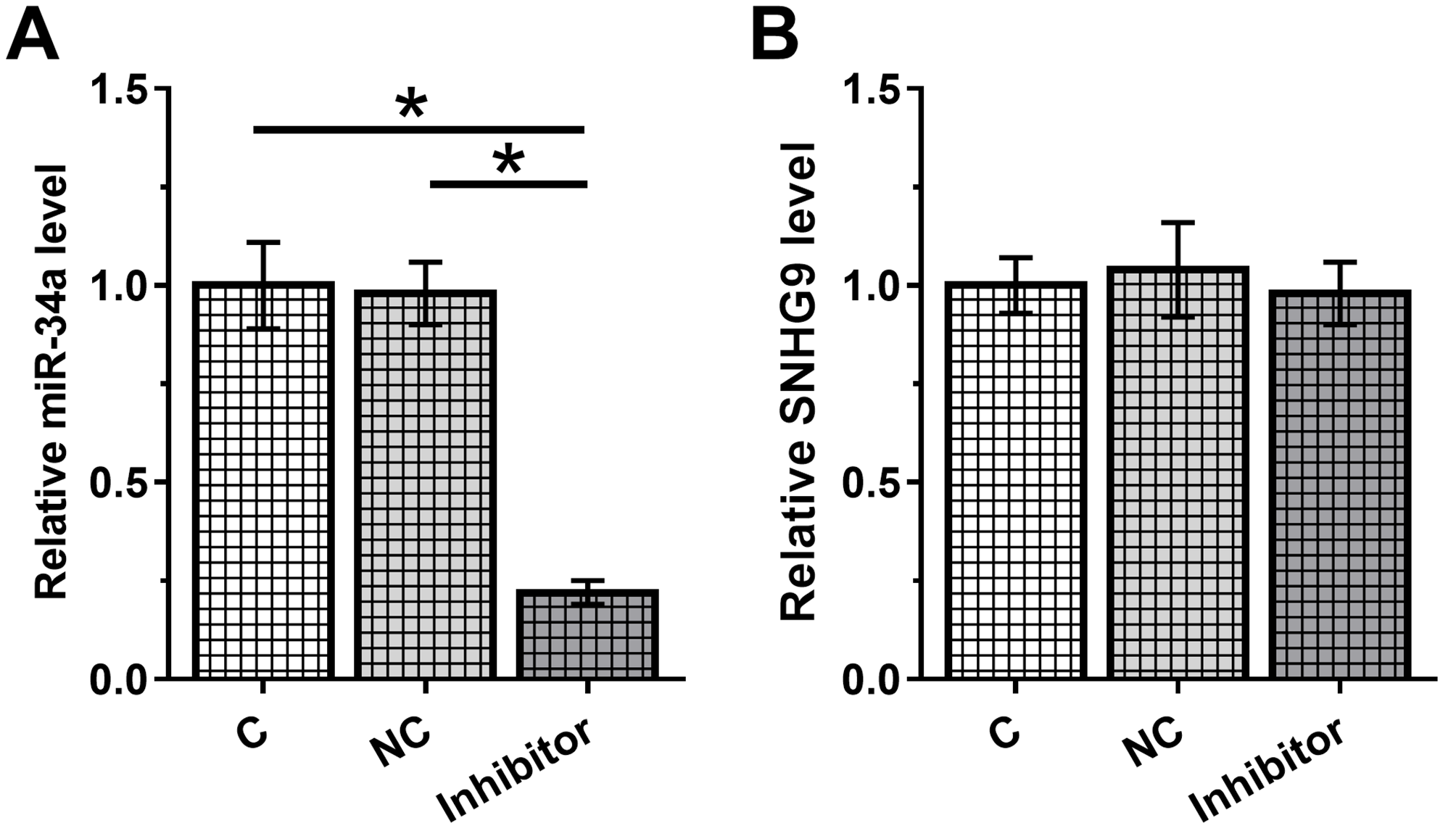

Supplement: Supplementary file 1 — Additional file 1: Figure S1. Inhibition of miR-34a did not affect the expression of SNHG9 in chondrocytes from OA patients. Inhibition of miR-34a in chondrocytes from OA patients was also performed (A). Inhibition of miR-34a also did not affect the expression of SNHG9 (B). All experiments were performed in triplicate manner and mean values were calculated and presented. *, p < 0.05. [file 12891_2020_3497_MOESM1_ESM.tif]

**Supplemental Figure 2 Full gel of figure 3D**


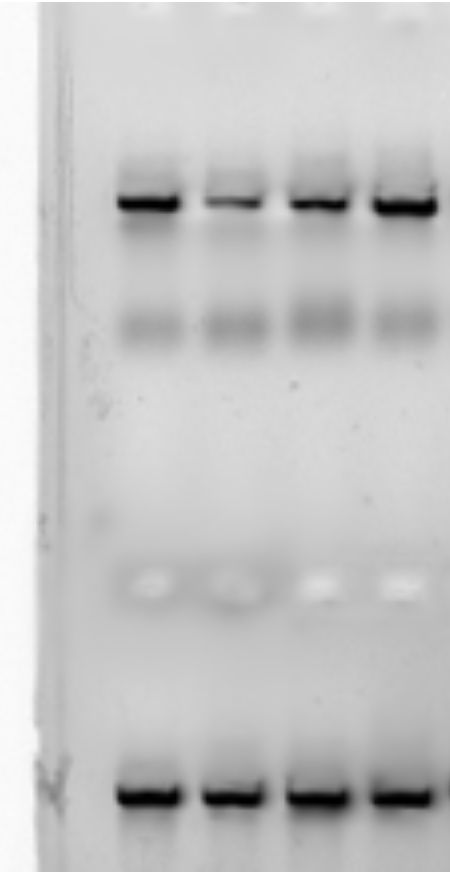

Supplement: Supplementary file 2 — Additional file 2: Figure S2. Full gel of Fig. 3d [file 12891_2020_3497_MOESM2_ESM.docx]
